# Exploring Mechanisms in COPD: Time for Biomarker Reappraisal?

**DOI:** 10.3390/jcm12216729

**Published:** 2023-10-25

**Authors:** Ilektra Voulgareli, Petros Bakakos, Stelios Loukides

**Affiliations:** 1 2nd Respiratory Medicine Department, Attikon University Hospital, Medical School, National and Kapodistrian University of Athens, 12462 Athens, Greece; ilektravoul@gmail.com; 2 1st Respiratory Medicine Department, Athens Chest Hospital Sotiria, Medical School, National and Kapodistrian University of Athens, 12462 Athens, Greece; pbakakos@med.uoa.gr

Chronic obstructive pulmonary disease (COPD) is a widespread condition often overlooked in diagnosis [1]. The intricate underlying mechanisms contribute to a wide range of clinical manifestations, posing challenges to accurate diagnosis and treatment. Given the diversity within COPD, the aim of this editorial was to identify markers that help to examine the distinct endotype and phenotype for each individual patient [1].

Systemic inflammation and oxidative stress contribute to COPD’s development [2]. Interestingly, serum albumin, which is an essential marker of malnutrition, possesses antioxidant properties as a negative acute-phase protein [2]. In patients with stable COPD, there is a notable reduction in serum albumin levels when compared to non-COPD counterparts [2]. This observation suggests an insufficiency in systemic anti-inflammatory and antioxidant defense mechanisms within the context of COPD [2], especially during exacerbations [3]. Moreover, not only serum albumin but also microalbuminuria, indicative of endothelial dysfunction, may serve as a pertinent inflammatory marker for potential systemic consequences of COPD as Romundstad et al. [4] posited the presence of a positive correlation between microalbuminuria and mortality in COPD individuals. Within COPD, mitochondria-derived reactive oxygen species (mtROS) are discharged from activated inflammatory cells or structural cells like epithelial, endothelial, or smooth muscle cells, signifying an adaptive response to the situation [3]. Furthermore, these reactive oxygen species (ROS) activate transcription factors such as nuclear factor-kappa B (NFkB). This leads to the release of pro-inflammatory mediators such as IL 1-like cytokines [3]. It is worth noting that the levels of IL-1β show an increase in the lungs of smoking-related COPD patients, suggesting potential involvement of the inflammasome [3].

Likewise, Macrophage Scavenger Receptor (MSR1) is a gene stimulated by hypoxia that has been linked to the removal of pathogens and apoptotic cells [5]. It has also been identified as a potential gene associated with COPD [5,6]. Various studies have highlighted that the processes leading to the accumulation of macrophages expressing MSR1 in the lungs might contribute to the development of severe emphysema [6].

Hence, extracellular heat shock protein 70 (eHsp70) appears to play a role in modulating immune responses within the context of COPD [1], as it seems to control the production of chemokines and the phosphorylation of EGFR in bronchial epithelial cells [7]. In COPD patients, the concentration of eHsp70 is higher compared to control subjects [1,8]. Moreover, this concentration increases in direct correlation with the severity of airflow limitation, the burden of symptoms, and the history of exacerbations [1]. Notably, eHsp70 levels exhibit a negative correlation with key lung function parameters such as forced expiratory volume in one second (FEV1) and the FEV1/forced vital capacity (FVC) ratio [1].

Furthermore, the prevailing impression is that airway inflammation in COPD primarily arises from Type1 immune responses, contributing to the obstructive nature of the disease [9]. However, a distinct aspect of Type2 inflammation has emerged in certain COPD patients, which is evident during stable periods and exacerbations [9]. Interestingly, gene expression analyses have identified a subset of COPD patients whose airways exhibit Type2 inflammation patterns. These individuals seem to respond favorably to inhaled corticosteroid (ICS) treatment [9]. Researchers are actively exploring Type2 inflammation biomarkers in COPD, such as sputum and blood eosinophils, exhaled nitric oxide fraction, and atopy [9]. A connection between FeNO levels and sputum eosinophil counts in stable COPD patients has been observed [10], as well as in COPD patients during exacerbations and at discharge following an exacerbation [11]. In a substantial prospective observational study involving 226 COPD patients, a relationship was identified between elevated FeNO levels and the likelihood of experiencing COPD exacerbations [12]. However, a study by Liu et al. [13] showed that concurrently elevated FeNO levels and elevated blood eosinophil counts have a protective effect on the progress of the disease, leading to a reduced occurrence and frequency of acute exacerbations. Besides, Kostikas et al. [14] found that patients whose cell count was below 50 cells/μL experienced increased mortality rates at both the 30-day and 1-year marks and that patients with cell counts of 150 cells/μL or more who received inhaled corticosteroids based on physicians’ recommendations showed better prevention of exacerbations during the follow-up period.

In addition, COPD is marked by continuously rising mortality rates, especially during exacerbations [15]. Recent investigations have shed light on the relationship between soluble suppression of tumorigenicity 2 (sST2) and mortality in COPD [15]. sST2 has emerged as an independent prognostic factor for all-cause mortality in COPD patients [15]. Moreover, evidence also points toward the potential significance of routine hematological parameters, such as Red cell Distribution Width (RDW), as it is reported that there are substantial connections between RDW and factors like disease presence, severity, outcomes (including mortality and hospital readmission), and pertinent clinical parameters (such as right heart failure and pulmonary arterial hypertension [16]. Especially, a study showed that the Red cell Distribution Width–albumin ratio was linked to hospital mortality in ICU-admitted COPD patients [17]. Furthermore, the Neutrophil-to-Lymphocyte Ratio (NLR), a cell inflammatory index also derived easily from routine hematological parameters, has been increasingly investigated as a diagnostic and prognostic biomarker in the context of COPD [18]. Generally, it is shown that there is a notable increase in the values of NLR in COPD patients experiencing an exacerbation when compared to stable COPD patients [19]. Also, it has been found that within 24–48 h of hospital admission, the measured NLR has shown a significant association with the risk of various adverse events during hospitalization, particularly short-term mortality up to 90 days in patients with a COPD exacerbation [18]. The above was confirmed by another study by Karkra et al. [20], which indicated that among individuals experiencing acute exacerbations of COPD, there was a statistically significant correlation between NLR and mortality.

Given this increasing evidence, there is a clear necessity to acquire fresh perspectives on the pathophysiology of COPD and examine potential biomarkers that can offer enhanced insights into different aspects of disease assessment, such as exacerbations, lung function decline, quality of life, and even mortality. All of the above-driven mechanisms may also provide an ideal environment for creating new treatment regimes and strategies.
